# Are Tanzanian patients attending public facilities or private retailers more likely to adhere to artemisinin-based combination therapy?

**DOI:** 10.1186/s12936-015-0602-x

**Published:** 2015-02-19

**Authors:** Katia Bruxvoort, Admirabilis Kalolella, Matthew Cairns, Charles Festo, Mitya Kenani, Peter Lyaruu, S Patrick Kachur, David Schellenberg, Catherine Goodman

**Affiliations:** London School of Hygiene and Tropical Medicine, London, UK; Ifakara Health Institute, Dar es Salaam, Tanzania; Malaria Branch, Centers for Disease Control and Prevention, Atlanta, USA

**Keywords:** Malaria, ACT, Adherence, Public health facilities, Private sector, ADDOs

## Abstract

**Background:**

Artemisinin combination therapy (ACT) is first-line treatment for malaria in most endemic countries and is increasingly available in the private sector. Most studies on ACT adherence have been conducted in the public sector, with minimal data from private retailers.

**Methods:**

Parallel studies were conducted in Tanzania, in which patients obtaining artemether-lumefantrine (AL) at 40 randomly selected public health facilities and 37 accredited drug dispensing outlets (ADDOs) were visited at home and questioned about doses taken. The effect of sector on adherence, controlling for potential confounders was assessed using logistic regression with a random effect for outlet.

**Results:**

Of 572 health facility patients and 450 ADDO patients, 74.5% (95% CI: 69.8, 78.8) and 69.8% (95% CI: 64.6, 74.5), respectively, completed treatment and 46.0% (95% CI: 40.9, 51.2) and 34.8% (95% CI: 30.1, 39.8) took each dose at the correct time (‘timely completion’). ADDO patients were wealthier, more educated, older, sought care later in the day, and were less likely to test positive for malaria than health facility patients. Controlling for patient characteristics, the adjusted odds of completed treatment and of timely completion for ADDO patients were 0.65 (95% CI: 0.43, 1.00) and 0.69 (95% CI: 0.47, 1.01) times that of health facility patients. Higher socio-economic status was associated with both adherence measures. Higher education was associated with completed treatment (adjusted OR = 1.68, 95% CI: 1.20, 2.36); obtaining AL in the evening was associated with timely completion (adjusted OR = 0.35, 95% CI: 0.19, 0.64). Factors associated with adherence in each sector were examined separately. In both sectors, recalling correct instructions was positively associated with both adherence measures. In health facility patients, but not ADDO patients, taking the first dose of AL at the outlet was associated with timely completion (adjusted OR = 2.11, 95% CI: 1.46, 3.04).

**Conclusion:**

When controlling for patient characteristics, there was some evidence that the adjusted odds of adherence for ADDO patients was lower than that for public health facility patients. Better understanding is needed of which patient care aspects are most important for adherence, including the role of effective provision of advice.

**Electronic supplementary material:**

The online version of this article (doi:10.1186/s12936-015-0602-x) contains supplementary material, which is available to authorized users.

## Background

As artemisinin-based combination therapy (ACT) for malaria becomes widely available, patient adherence to the full course of treatment is increasingly important to ensure positive clinical outcomes and minimize the selection of drug-resistant parasites [1-3]. ACT is the first-line treatment for *Plasmodium falciparum* malaria in most malaria-endemic countries and has become increasingly available in the private retail sector, where many patients seek care for malaria [4-9].

Estimates of patient adherence to ACT range from 39 to 100%, reflecting both variation in patient characteristics, interaction with providers, study settings, differences in study procedures, and methods of assessing adherence [10,11]. The vast majority of studies designed to measure adherence have been conducted in the public sector, while a few studies have used household survey data to assess adherence from mixed community sources [12-17]. Only one study had specifically addressed patient adherence to ACT obtained in the private sector. This study, conducted in a convenience sample of four shops in a single Ugandan district where subsidized ACT had been made available through a pilot programme, reported that 66% of patients seeking treatment completed the full course of ACT [18].

As access to ACT increases, there is a need to understand levels and determinants of patient adherence in both public and private sectors, in order to design and target appropriate interventions. Patients seeking care in the private retail sector may have different characteristics (e.g., age, socio-economic status, illness severity, etc.) than patients who seek care in public health facilities [7,19,20], though some patients seek care in both sectors. Similarly, provider characteristics (e.g., training, communication, reputation, costs, drug availability, motivation, etc.) may also vary and affect where patients seek care [7,21]. It is unclear how these differences affect patient adherence.

In Tanzania, artemether-lumefantrine (AL) for treatment of uncomplicated malaria was first rolled out to public health facilities in 2006. The recommended treatment regimen is six doses of AL over three days, with one to four tablets (20 mg artemether/120 mg lumefantrine) per dose depending on the patient's weight/age band. National guidelines state that the first dose should be taken under observation of the dispenser, the second dose eight hours after the first dose, and the remaining doses morning and evening of the second and third days [22]. Treatment at public health facilities for children under five years of age and pregnant women is intended to be free of charge, but this policy is not always followed [20].

Treatment for malaria in the private sector in Tanzania is sought at private health facilities, pharmacies, drug shops, and general stores. More than two-thirds of anti-malarial drug sales from private for-profit providers occur in drug shops [23], many of which have been upgraded through the accredited drug dispensing outlet (ADDO) programme. ADDOs were first piloted in 2003 in order to improve availability, quality, and affordability of pharmaceutical services in rural and peri-urban areas without pharmacies. ADDOs have now been rolled-out nationwide, with an estimated 9,000 ADDOs serving the 25 regions of mainland Tanzania [24]. The ADDO programme involves multiple components including training, accreditation, and regulatory oversight by trained inspectors [24-26]. Dispensers are required to have a health qualification of a nurse assistant or higher and must attend a six-week training course on topics such as business practices, regulations, record-keeping, use of ADDO-approved medicines, dispenser ethics and communication skills. ADDOs are allowed to sell a limited number of prescription-only drugs, including an approved list of antibiotics and anti-malarial drugs. Availability of ACT was limited in ADDOs until the implementation of the Affordable Medicines Facility- malaria (AMFm) in 2010, which led to a significant reduction in price and a corresponding increase in availability [23,27].

This paper reports results of two parallel, contemporaneous studies in southern Tanzania to compare patient adherence to ACT obtained in public health facilities with adherence to ACT obtained from ADDOs in the same area and to examine factors associated with adherence in each sector.

## Methods

### Study setting

The studies were conducted in Mtwara, a rural region in southeastern Tanzania with more than a third of the population in the lowest national wealth quintile [28]. In 2012, malaria prevalence in Mtwara in patients of all ages seeking care for febrile illness was 15% among patients attending ADDOs and 32% among patients attending public health facilities [29,30]. Conversion of drug shops to ADDOs in Mtwara commenced in 2006, with all drug shops in Mtwara officially required to have upgraded to ADDO status by 2011. At the time of the study, some shops had not yet paid fees, received training, or been visited by inspectors but were tolerated as ‘prospective ADDOs’ (in this paper the term ADDOs is used to include both accredited outlets and ‘prospective ADDOs’). To support the increased availability of ACT in ADDOs, the government offered a one-day training that included treatment of malaria with ACT to dispensers with previous nursing training in Mtwara in August 2011.

### Study design

In health facilities, a descriptive study was conducted to assess patient adherence to AL. The study in ADDOs was designed as part of a cluster-randomized trial to evaluate a text message intervention to improve dispenser knowledge of advice to provide to patients obtaining AL. Details of the intervention and results of the trial are presented separately [31]. Only data from the control arm, which did not receive any intervention, are presented here.

### Sample size calculations

The target sample size for ADDO patients was based on the text message intervention trial [31], and a similar sample size was desired for public health facilities within the constraints of study resources. Based on adherence previously reported among patients obtaining ACT at public health facilities in Tanzania, which ranged from 75 to 98% [32-34], adherence among patients attending public health facilities was expected to be 75%, with lower adherence among patients attending ADDOs (60%). Assuming a hypothesized design effect of 2.5 and 20% loss to follow-up, 448 registered patients from 36 outlets in each sector (approximately 12 to 13 patients per outlet) would have 80% power to detect this difference with 95% confidence. Given the lower design effect observed in the study (1.5) and more interviewed patients than anticipated, the study was powered to detect a 10 percentage point difference between sectors, but was not able to detect if a smaller difference in completion rates between sectors was due to random error.

### Selection of outlets

A list of all public dispensaries and health centres in Mtwara, excluding district hospitals, was compiled by visiting each district and interviewing the district medical officers. The health facilities were randomly ordered, and the first 40 health facilities were selected. Sampling of ADDOs was based on a census in Mtwara of all ADDOs, including prospective ADDOs, conducted prior to the text message intervention trial. From this list, ADDOs were selected sequentially at random, with all ADDOs within 400 m of a selected ADDO, or any ADDO where staff from a selected ADDO also worked, removed from the sampling frame. Forty ADDOs were randomized to the control arm [31].

### Study procedures

From September through November 2012, dispensers at selected public health facilities and ADDOs were visited by study supervisors and given a standard introduction about study objectives. In order to limit patients’ awareness of the primary interest in adherence, which could have led to a biased assessment, dispensers were told the focus was how patients chose to treat fever and that some, but not all, patients would be visited at their homes. Dispensers were asked to fill out a registration form for all patients dispensed any treatment for fever, including day and time of the visit, the patient’s name, drugs dispensed, and a description of where the patient lived. Dispensers were provided with blister packs of AL to be dispensed in public health facilities to patients prescribed ACT, and in ADDOs to patients indicating an intention to purchase treatment for malaria. Study staff visited outlets every day to check and collect registration forms. While the intention had been to register 12 patients obtaining ACT for one week per outlet, the protocol was adjusted due to low attendance at some outlets to register all patients obtaining ACT for two to three weeks per outlet.

Eligible patients who obtained AL were identified from the registration forms and assigned patient identification numbers. Patients were visited at their homes three days later (day 4), and all attempts to locate and interview patients were recorded. Where written informed consent was given, patients or their caregivers were asked about demographic and socio-economic characteristics, treatment-seeking history, symptoms, and detailed information about each dose of AL taken. Patients were asked if dispensers provided each of several aspects of advice on AL (e.g., number of pills to take per dose, when to take second dose, etc.), and if so, what advice was given. Blister packs were also requested for a pill count. Since the *P. falciparum* histidine-rich protein II (HRP-2) persists in the blood following treatment, HRP-2-based malaria rapid diagnostic tests (mRDTs) (Pf-specific from ICT Diagnostics, Cape Town, South Africa) were conducted to indicate infection prior to treatment. Blood smears were collected to detect infection at the time of interview. Blood smears were stained in the field and transported to the Ifakara Health Institute, where they were double-read by two microscopists blinded to results from each other and the mRDT, with discrepant readings resolved by a third microscopist.

Adherence was defined in two ways [10]. Patients were considered to have verified completed treatment if they reported taking all doses by the time of the interview and, when available, a pill count verified that no pills remained in the blister pack. Where blister packs were not available, self-report alone determined if patients completed treatment. The second, more stringent definition included a time component, based on patient reports of the time each dose was taken using the Swahili times of day: *alfajiri* (early morning), *asubuhi* (morning), *mchana* (afternoon), *jioni* (evening), *usiku* (night), and *usiku sana* (late night). Patients were considered to have verified timely completion if they took the correct number of pills for each dose, and took the second dose at the Swahili time of day corresponding with eight hours after the first dose, followed by taking each of the remaining doses at the Swahili time of day corresponding with 12 hours after the previous dose, verified when possible by the absence of pills in the blister pack. The terms completed treatment and timely completion are hereafter used to refer to these definitions.

### Data entry and analysis

All patient and dispenser interview data were collected using personal digital assistants, and data extracted from study forms (census, registration and follow-up forms) were double entered into Microsoft Access databases. Data were analysed in Stata 11.0 (Stata Corporation, College Station, USA). Robust standard errors were used for percentages and 95% confidence intervals, with p-values reported for the Pearson design-based F test. Wealth quintiles describing socio-economic status were assigned to patients based on standard Demographic and Health Survey variables, using principal components analysis of the pooled sample of public health facility and ADDO patients [28]. Two analyses of factors associated with adherence were conducted: (i) a comparison of adherence between the public and private sectors controlling for patient characteristics; and, (ii) an analysis within each sector exploring the association of adherence with factors related to the care received at the outlet and patient characteristics.

### Comparison of adherence between sectors

In the analysis on the impact of sector on patient adherence, random effects logistic regression was used for both completed treatment and timely completion to compare the odds of adherence between private sector ADDO patients and public sector health facility patients, adjusting for patient characteristics identified *a priori* (age group, education and time between obtaining AL and interview) or those that made important changes to the odds ratio for sector in bivariate models. In this analysis, no adjustment was made for variables related to care received at the outlet (e.g., taking first dose at outlet, recalling correct instructions on how to take AL, etc.), as these factors might mediate the effect of sector (i.e., lie on the causal pathway between sector and adherence).

### Factors associated with adherence within sectors

Within each sector, the association of variables related to care received at the outlet with 1) completed treatment, and, 2) timely completion was explored in univariate and multivariate models. Logistic regression with robust standard errors was used, as checks showed that the quadrature approximations for random effects models were not reliable. Patient characteristics and care-related variables that were associated with completed treatment or timely completion in either sector in unadjusted analyses were included in all four multivariate models.

### Ethics

All questionnaires, consent forms and other study documents were translated into Swahili and piloted prior to use. Written informed consent was collected from dispensers prior to census, patient registration, and interview and from patients or their caregivers prior to interview. The study protocol was approved by the ethical review boards of Ifakara Health Institute and London School of Hygiene and Tropical Medicine. CDC advisors provided technical assistance in design and analysis but were not engaged in data collection and did not have access to personal identifiers.

## Results

### Patient characteristics, care received, and status at interview

Data were collected from patients obtaining AL at all 40 selected health facilities and 37 of 40 selected ADDOs, with three ADDOs closed or refusing to participate. Of 604 registered health facility patients obtaining AL (median = 16 patients per outlet, range two to 32), 572 patients (95%) were interviewed. From ADDOs, 537 patients obtaining AL were registered (median = 17 patients per outlet, range one to 29), and 450 patients (84%) were interviewed. The most common reasons in both sectors for non-completion of interviews were not locating the patient’s home (38% at public facilities and 43% at ADDOs), or the patient having travelled out of the study region (28 and 16%).

Characteristics of patients differed between sectors (Table 1). ADDO patients were more likely to be male and older than those attending public health facilities. ADDO patients/caregivers were wealthier, with 32% in the least poor wealth quintile compared to 10% of public health facility patients, and were more likely to have finished primary school (72 *vs* 58%, p = 0.007). Over a third of patients in both sectors had previously sought care for their illness episode, many of whom had gone to a general store/kiosk or taken drugs stored at home or from a neighbour. Reported symptoms were similar across outlet type, except more patients from public health facilities had experienced respiratory symptoms (14 *vs* 7.5%, p = 0.007) and more ADDO patients had experienced body pain (30 *vs* 15%, p < 0.0001) or fatigue (18 *vs* 10%, p = 0.003), with few patients in either sector reporting convulsions or other signs of severe disease. ADDOs were much more likely than public health facilities to be located in urban areas, but outlets in both sectors were attended mostly by patients living within 2.5 km (about half-an-hour walk) from the outlet. ADDO patients were also more likely to have obtained AL later in the day, reflecting the fact that most public health facilities in Mtwara close for outpatient services by mid-afternoon, while ADDOs often stay open through the evening. Because of this, the day-4 interview was more likely to occur earlier (between 60–67 hours from the time the drug was obtained) for ADDO patients than for public health facility patients (25 *vs* 6%).

**Table 1:** **Patient characteristics by sector (%, 95% CI)**

	**Public health facilities (N = 572 patients from 40 outlets)**	**Private ADDOs (N = 450 patients from 37 outlets)**	**p-value**
Male	43.2 (39.9, 46.5)	53.1 (46.8, 59.4)	0.007
*Age* ^1^			
Under 3 years	42.5 (37.4, 47.8)	18.2 (14.5, 22.6)	
3 years to under 8 years	28.2 (23.5, 33.3)	23.1 (18.8, 28.0)	
8 years to under 12 years	6.6 (4.3, 10.0)	9.1 (6.9, 12.0)	
12 years and above	22.7 (18.7, 27.3)	49.6 (43.0, 56.2)	<0.0001
Patient (or caregiver if patient below age 12) completed primary school^2^	58.2 (50.7, 65.4)	71.8 (65.2, 77.6)	0.007
*Socio-economic status* ^*3*^			
1^st^ quintile (most poor)	27.9 (23.1, 33.2)	10.2 (7.6, 13.5)	
2^nd^ quintile	24.5 (20.5, 29.1)	14.2 (10.1, 19.8)	
3^rd^ quintile	20.0 (16.5, 23.9)	20.2 (15.4, 26.1)	
4^th^ quintile	17.3 (13.1, 22.6)	23.3 (19.5, 27.6)	
5^th^ quintile (least poor)	10.3 (7.4, 14.4)	32.1 (23.3, 42.2)	<0.0001
Slept under net the night before the interview	73.6 (68.9, 77.8)	71.4 (65.3, 76.9)	0.6
Sought care for this episode prior to attending outlet	36.4 (31.9, 41.2)	37.8 (31.6, 44.4)	0.7
Sought care at outlet within two days of fever onset^4^	77.5 (73.5, 81.1)	72.0 (67.8, 75.9)	0.051
*Symptoms*			
Fever or headache	94.1 (91.3, 96.0)	91.1 (87.6, 93.7)	0.1
Respiratory	14.0 (10.5, 18.4)	7.5 (5.3, 10.7)	0.007
Stomach upset	53.5 (47.7, 59.2)	48.9 (42.3 55.6)	0.3
Body/joint pain	15.4 (12.2, 19.1)	30.4 (25.2, 36.2)	<0.0001
Fatigue	10.1 (7.5, 13.6)	18.0 (14.2, 22.5)	0.003
Convulsions	2.5 (1.6, 3.9)	0.4 (0.1, 1.7)	0.007
Other^5^	12.4 (9.6, 15.9)	10.0 (7.5, 13.1)	0.2
Attended an outlet in an urban ward	13.1 (5.1, 29.6)	68.4 (48.3, 83.4)	<0.0001
Distance of 2.5 km or less from home to outlet (by GPS coordinates)^6^	69.4 (62.6, 75.4)	71.4 (58.5, 81.5)	0.8
*Time of day drug was obtained*			
Morning	77.1 (71.9, 81.6)	44.7 (38.2, 51.3)	
Afternoon	18.7 (14.6, 23.6)	26.9 (22.2, 32.2)	
Evening	4.2 (2.5, 7.0)	28.4 (21.6, 36.4)	<0.0001
*Time between obtaining AL and interview (hours)* ^7^			
60-67	5.8 (3.8, 8.6)	24.8 (20.7, 29.3)	
68-72	47.0 (41.7, 52.4)	37.4 (33.1, 41.9)	
73-84	32.5 (28.5, 36.7)	21.6 (16.9, 27.2)	
85 or more	14.7 (11.4, 18.8)	16.2 (12.1, 21.4)	<0.0001

^1^Age categories based on recommended age breakdown for AL blister packs in Tanzania.

^2^Caregiver education missing for five public health facility patients.

^3^Wealth quintiles pooled for public health facilities and ADDOs using principal component analysis of sampled patients based on standard Demographic and Health Survey variables. Data missing for one public health facility patient.

^4^Number of days since illness onset missing for 3 public health facility patients and 11 ADDO patients.

^5^Includes dizziness, crying/fussiness, startling (*kustukastuka*), sleep-talking (*kuweweseka*), worms, fast heart rate, stays in sun, red/inflamed eyes, and sores/ulcers.

^6^GPS data missing from 30 public health facility patients and 52 ADDO patients.

^7^Rounded to nearest hour. Data missing for 15 public health facility patients and 6 ADDO patients.

Table 2 compares care received at the outlet and patient status at interview for each sector. Half of the public health facility patients reported being tested for malaria, while 11% reported being tested in ADDOs, where diagnostic tests for malaria had not been officially introduced. Public health facility patients were slightly more likely to be told a diagnosis (64 *vs* 54%, p = 0.051) and more likely to take the first dose of AL immediately at the outlet (41 *vs* 10%, p < 0.0001), while ADDO patients were more likely to pay for AL (98 *vs* 29%, p < 0.0001). Similar percentages of patients using outlets in both sectors reported receiving advice on how to take AL from the dispenser, with 60% able to recall correct instructions on the number of pills per dose, number of doses per day, and number of days to take AL. Approximately 60% of patients in both sectors reported being told to take the second dose of AL after eight hours and to take each dose with food or milk. Public health facility patients were more likely to recall being told to finish all doses even if feeling better (78 *vs* 63%, p = 0.0006). However, less than 5% of patients treated in either sector reported being advised on possible side effects or on what to do in case of vomiting within half-an-hour of taking a dose.

**Table 2:** **Care received at outlet and patient status at interview by sector (%, 95% CI)**

	**Public health facilities (N = 572 patients from 40 outlets)**	**Private ADDOs (N = 450 patients from 37 outlets)**	**p-value**
*Treatment received at outlet*			
Tested for malaria	54.4 (40.3, 67.9)	11.1 (8.0, 15.4)	<0.0001
Told diagnosis	64.1 (56.3, 71.1)	53.5 (46.1, 60.8)	0.051
Obtained correct blister pack for age^1^	78.9 (74.7, 82.5)	83.1 (78.7, 86.8)	0.1
Paid for AL	28.7 (24.0, 34.0)	97.8 (96.1, 98.7)	<0.0001
Took first dose of AL at outlet	40.7 (29.8, 52.7)	9.6 (6.3, 14.2)	<0.0001
*Recall of instructions received from dispenser*			
Recalled correct instructions given by dispenser on the number of pills per dose, number of doses, and number of days to take AL	60.8 (56.4, 65.2)	59.3 (53.7, 64.7)	0.7
Recalled that dispenser used packaging as a visual aid to explain how to take AL	85.6 (81.9, 88.6)	82.9 (77.9, 86.9)	0.3
Reported being told to take the second dose of AL eight hours after the first dose	58.0 (51.3, 64.5)	63.3 (57.9, 68.4)	0.2
Reported being told to take AL with food or milk	63.8 (57.9, 69.3)	61.8 (54.6, 68.5)	0.7
Reported being told to complete all doses of AL even if feeling better	77.9 (73.5, 81.7)	63.3 (55.7, 70.4)	0.0006
Reported being told to take a replacement dose in case of vomiting within half hour of taking a dose	1.9 (1.1, 3.5)	2.4 (1.3, 4.6)	0.6
Reported being told about possible side effects	2.3 (1.3, 4.0)	3.0 (1.6, 5.6)	0.6
*Health status at interview*			
Reported current fever at time of interview	13.7 (10.5, 17.7)	14.7 (11.1, 19.1)	0.7
Could play or work at time of interview	92.6 (89.2, 95.1)	92.4 (89.7, 94.5)	0.9
Tested positive by mRDT at interview^2^	49.6 (39.7, 59.5)	27.9 (20.7, 36.6)	0.001
Tested positive by blood smear collected at interview^3^	2.9 (1.7, 4.7)	1.4 (0.6, 3.0)	0.1
*Adherence to AL*			
Adherent by ‘verified completed treatment’^4^	74.6 (69.8, 78.8)	69.8 (64.6, 74.5)	0.2
Adherent by ‘verified timely completion’^5^	46.0 (40.9, 51.2)	34.8 (30.1, 39.8)	0.003

^1^Age categories based on recommended age breakdown for AL blister packs in Tanzania.

^2^mRDT data missing for 7 public health facility patients and 17 ADDO patients.

^3^Blood smear data missing for 15 public health facility patients and 18 ADDO patients.

^4^Patient completed all doses, verified by pill count when available. Data missing for 2 public health facility patients and 3 ADDO patients.

^5^Patient completed each dose at correct time with the correct number of pills per dose, verified by pill count when available. Data missing for 13 public health facility patients and 10 ADDO patients.

At the time of interview, approximately 14% of both public health facility and ADDO patients reported a current fever and 92% could play or work. More patients who had attended public health facilities tested positive by the mRDT performed by study staff during the interview (50% compared to 28% of ADDO patients, p = 0.001). However, by reference blood smear, indicating current infection status at the time of interview, only 2.9% of public health facility patients and 1.4% of ADDO patients were positive (p = 0.1).

### Comparison of adherence between sectors

Among public health facility patients, 74.5% (95% CI: 69.8, 78.8) completed treatment, compared with 69.8% (95% CI: 64.6, 74.5) among ADDO patients (p = 0.2). Timely completion was much lower and differed between sectors, with 46.0% (95% CI: 40.9, 51.2) of public health facility patients and 34.8% (95% CI: 30.1, 39.8) of ADDO patients taking the correct number of pills at the correct time of day for each dose (p = 0.003). Variables that made important differences to the odds ratio for sector in the bivariate models were wealth quintile, distance from home to outlet, and time of day AL was obtained, and these were included along with age group, patient/caregiver education, and time between obtaining AL and interview in the models comparing adherence between sectors. Patients in the two least poor wealth quintiles had higher adjusted odds of both measures of adherence compared to those in the poorest wealth quintile (Table 3). The adjusted odds of completed treatment for those who had finished primary school was 1.68 times that of patients who had not (95% CI: 1.20, 2.36; p = 0.003), but there was no evidence of an association with timely completion (aOR = 1.06, 95% CI: 0.77, 1.75; p = 0.9). Compared to patients who obtained AL in the morning, patients who obtained AL in the evening were similarly likely to complete treatment (aOR = 0.93, 95% CI: 0.50, 1.70; p = 0.8), but had much lower adjusted odds of timely completion (aOR = 0.35, 95% CI: 0.19, 0.64; p = 0.001). Furthermore, patients interviewed a longer time after obtaining AL (68–72 hours, 73–84 hours, and 85 hours or more) had higher adjusted odds of completed treatment and timely completion than patients visited 60–67 hours after obtaining AL (Table 3). When controlling for these patient characteristics, the adjusted odds of completed treatment for ADDO patients was 0.65 times the odds of completed treatment for public health facility patients (95% CI: 0.43, 1.00; p = 0.048), and the adjusted odds of timely completion for ADDO patients was 0.69 that of health facility patients (95% CI: 0.47, 1.01; p = 0.056).

**Table 3:** **Effect of sector on adherence controlling for potential confounders** ^**1**^

	**Verified completed treatment** ^**2**^		**Verified timely completion** ^**3**^	
	**Adjusted odds ratio**	**p-value**	**Adjusted odds ratio**	**p-value**
Attended ADDO vs. public health facility	0.65 (0.43, 1.00)	0.048	0.69 (0.47, 1.01)	0.056
*Age* ^4^				
Under 3 years (ref)	---	---	---	---
3 years to under 8 years	1.01 (0.67, 1.52)	0.9	0.88 (0.61, 1.28)	0.5
8 years to under 12 years	1.07 (0.57, 2.09)	0.8	1.12 (0.62, 2.01)	0.7
12 years and above	1.02 (0.56, 2.05)	0.8	0.87 (0.60, 1.27)	0.5
Patient (or caregiver if patient below age 12) completed primary school	1.68 (1.20, 2.36)	0.003	1.06 (0.77, 1.45)	0.9
*Socio-economic status* ^5^				
1^st^ quintile (most poor, ref)			---	---
2^nd^ quintile	0.98 (0.62, 1.57)	0.9	1.04 (0.66, 1.64)	0.9
3^rd^ quintile	1.17 (0.73, 1.88)	0.5	1.10 (0.70, 1.75)	0.7
4^th^ quintile	2.25 (1.33, 3.81)	0.003	1.64 (1.03, 2.65)	0.039
5^th^ quintile (least poor)	2.24 (1.28, 3.81)	0.005	2.34 (1.40, 3.93)	0.001
Distance from home to outlet within 2.5 km by GPS	1.30 (0.92, 1.85)	0.2	1.20 (0.87, 1.66)	0.3
*Time of day drug was obtained*				
Morning (ref)	---	---	---	---
Afternoon	0.96 (0.62, 1.47)	0.8	0.70 (0.48, 1.03)	0.070
Evening	0.93 (0.50, 1.70)	0.8	0.35 (0.19, 0.64)	0.001
*Time between obtaining AL and interview (hours)*				
60-67 (ref)	---	---	---	---
68-72	2.43 (1.39, 4.23)	0.002	1.46 (0.81, 2.63)	0.2
73-84	2.83 (1.49, 5.37)	0.001	1.92 (1.00, 3.66)	0.049
85 or more	6.44 (3.19, 13.01)	<0.001	2.61(1.37, 4.95)	0.003

^1^Number of observations = 912 (110 patients excluded from model due to missing data) and number of outlets = 77. Covariates are those presented in Table.

^2^Patient completed all doses, verified by pill count when available. Data missing for 5 patients.

^3^For each dose, patients took the correct number of pills at the correct time of day, verified by pill count when available. Data missing for 23 patients.

^4^Age categories based on recommended age breakdown for AL blister packs in Tanzania.

^5^Wealth quintiles pooled for public health facilities and ADDOs using principal component analysis of sampled patients based on standard Demographic and Health Survey variables.

### Factors associated with adherence within sectors

Unadjusted associations of patient characteristics and care received at the outlet with completed treatment and timely completion are presented for each sector in Additional files 1 and 2. Variables not associated with either adherence measure in either sector, such as patient sex, having sought previous treatment, report of specific symptoms, and paying for AL, were not included in the sector specific multivariate analyses shown in Table 4. In contrast with the analyses comparing the effect of sector on adherence, in these sector specific models there were no clear patterns of association between education and socioeconomic status and either measure of adherence. Similar to the previous analysis for the effect of sector, patients obtaining AL in the afternoon or in the evening had lower adjusted odds of timely completion than those obtaining AL in the morning, although this was the case only in public health facilities. In both public health facilities and ADDOs, longer time between obtaining AL and interview were again strongly associated with completed treatment. However, there was no evidence of an association with timely completion in public health facilities, and in ADDOs only being interviewed 72–84 hours after obtaining AL (not 68–72 hours or 85 hours or more) compared to 60–67 hours was associated with timely completion. In addition, public health facility patients, but not ADDO patients, sleeping under a bed net the night before the interview, having experienced fever symptoms, and living within 2.5 km of the outlet was associated with completed treatment, while seeking care within two days of fever onset was associated with timely completion.

**Table 4:** **Multivariate analyses of factors associated with adherence, by sector**

	**Verified completed treatment** ^**1**^				**Verified timely completion** ^**2**^			
	**Public health facilities (N = 572)** ^**3**^		**ADDOs (N = 450)** ^**4**^		**Public health facilities (N = 572)** ^**3**^		**ADDOs (N = 450)** ^**4**^	
	**Adjusted odds ratio (95% CI)**	**p-value**	**Adjusted odds ratio (95% CI)**	**p value**	**Adjusted odds ratio (95% CI)**	**p-value**	**Adjusted odds ratio (95% CI)**	**p-value**
*Age* ^5^								
Under 3 years (ref)	---	---	---	---	---	---	---	---
3 years to under 8 years	1.32 (0.76, 2.28)	0.3	0.69 (0.35, 1.36)	0.3	1.03 (0.60, 1.78)	0.9	0.51 (0.28, 0.93)	0.029
8 years to under 12 years	1.33 (0.49, 3.57)	0.6	0.97 (0.43, 2.16)	0.9	2.25 (0.74, 6.86)	0.2	0.75 (0.31, 1.82)	0.5
12 years and above	1.19 (0.62, 2.31)	0.6	0.94 (0.48, 1.84)	0.9	1.06 (0.59, 1.93)	0.8	0.69 (0.35, 1.38)	0.3
Patient (or caregiver if patient below age 12) completed primary school	1.16 (0.70, 1.91)	0.6	1.56 (1.00, 2.43)	0.050	0.88 (0.55, 1.40)	0.6	0.94 (0.54, 1.63)	0.8
*Socio-economic status* ^*6*^								
1^st^ quintile (most poor, ref)	---	---	---	---	---	---	---	---
2^nd^ quintile	0.97 (0.60, 1.57)	0.9	0.82 (0.38, 1.81)	0.6	0.93 (0.52, 1.64)	0.8	1.19 (0.62, 2.26)	0.6
3^rd^ quintile	1.04 (0.54, 1.98)	0.9	1.48 (0.72, 3.03)	0.3	0.70 (0.37, 1.34)	0.3	2.84 (1.15, 7.05)	0.024
4^th^ quintile	2.20 (1.09, 4.47)	0.038	1.75 (0.87, 3.51)	0.1	1.25 (0.64, 2.44)	0.5	1.82 (0.61, 5.45)	0.3
5^th^ quintile (least poor)	2.19 (0.81, 5.93)	0.2	1.99 (0.71, 5.54)	0.2	2.21 (1.01, 4.82)	0.046	2.47 (0.93, 6.55)	0.070
Slept under a bed net the night before the interview	1.60 (1.10, 2.34)	0.015	0.98 (0.59, 1.62)	0.9	1.23 (0.77, 1.96)	0.4	0.79 (0.50, 1.27)	0.3
Sought care within two days of fever onset	1.01 (0.60, 1.72)	0.9	1.16 (0.63, 2.13)	0.6	1.61 (0.99, 2.61)	0.056	1.11 (0.59, 2.09)	0.8
Fever symptoms	3.38 (1.21, 9.47)	0.020	1.18 (0.56, 2.47)	0.7	1.65 (0.64, 4.25)	0.3	0.86 (0.47, 1.58)	0.6
Distance from home to outlet within 2.5 km^7^	1.67 (1.05, 2.65)	0.031	0.76 (0.48, 1.20)	0.2	1.14 (0.68, 1.91)	0.6	1.09 (0.65, 1.82)	0.8
*Time of day drug was obtained*								
Morning (ref)	---	---	---	---	---	---	---	---
Afternoon	1.11 (0.62, 2.01)	0.7	1.12 (0.52, 2.38)	0.8	0.55 (0.31, 0.97)	0.038	1.04 (0.57, 1.88)	0.9
Evening	0.81 (0.22, 3.04)	0.7	0.95 (0.44, 2.05)	0.9	0.10 (0.02, 47.6)	0.004	0.69 (0.31, 1.53)	0.4
*Time between obtaining AL and interview (hours)*								
60-67 (ref)	---	---	---	---	---	---	---	---
68-72	2.37 (0.87, 6.51)	0.093	2.80 (1.35, 5.82)	0.006	1.47 (0.44, 4.95)	0.5	1.55 (0.71, 3.36)	0.3
73-84	3.64 (1.31, 10.08)	0.013	2.73 (1.12, 6.63)	0.027	2.03 (0.56, 7.29)	0.3	2.94 (1.20, 7.20)	0.018
85 or more	5.59 (1.66, 18.79)	0.005	5.94 (2.70, 13.06)	<0.001	2.72 (0.75, 9.84)	0.1	2.09 (0.85, 5.13)	0.1
Tested for malaria at outlet	1.30 (0.82, 2.04)	0.3	0.39 (0.17, 0.85)	0.018	1.47 (0.96, 2.26)	0.078	0.48 (0.24, 0.97)	0.041
Took first dose of AL at outlet	1.05 (0.71, 1.55)	0.8	1.34 (0.60, 3.01)	0.5	2.11 (1.46, 3.04)	<0.001	1.33 (0.57, 3.13)	0.5
Recalled correct instructions given by dispenser on the number of pills per dose, number of doses, and number of days to take AL	4.04 (2.59, 6.31)	<0.001	2.98 (2.03, 4.37)	<0.001	6.09 (3.71, 10.02)	<0.001	2.51 (1.41, 4.45)	0.002
Recalled that dispenser used packaging as a visual aid to explain how to take AL	1.33 (0.75, 2.36)	0.3	1.28 (0.62, 2.66)	0.5	1.85 (1.09, 3.11)	0.022	1.40 (0.73, 2.67)	0.3
Reported being told to take the second dose of AL eight hours after the first dose	1.15 (0.74, 1.79)	0.5	1.28 (0.87, 1.89)	0.2	0.85 (0.52, 1.38)	0.5	1.77 (1.12, 2.80)	0.015
Reported being told to complete all doses of AL even if feeling better	0.96 (0.54, 1.71)	0.9	1.02 (0.61, 1.70)	0.9	0.44 (0.28, 0.70)	0.001	1.05 (0.73, 1.50)	0.8

^1^Patient completed all doses, verified by pill count when available. Data missing for 2 public health facility patients and 3 ADDO patients.

^2^Patient completed each dose at correct time with the correct number of pills per dose, verified by pill count when available. Data missing for 13 public health facility patients and 10 ADDO patients.

^3^Standard errors adjusted for 37 clusters.

^4^Standard errors adjusted for 40 clusters.

^5^Age categories based on recommended age breakdown for AL blister packs in Tanzania.

^6^Wealth quintiles pooled for public health facilities and ADDOs using principal component analysis of sampled patients based on standard Demographic and Health Survey variables.

^7^Based on GPS coordinates. Data missing from 30 public health facility patients and 52 ADDO patients.

Factors related to care received at the outlet varied by sector in their associations with both adherence measures (Table 4). In the public sector, reporting being tested for malaria at the outlet was not associated with completed treatment (aOR = 1.30, 95% CI: 0.82, 2.04; p = 0.3), but there was weak evidence of an association with timely completion (aOR = 1.47, 95% CI: 0.96-2.29; p = 0.078). Among ADDO patients, however, the adjusted odds of completed treatment and timely completion were lower for those who reported being tested compared to those who did not report being tested (aOR = 0.39, 95% CI: 0.17-0.85; p = 0.018 and aOR = 0.48, 95% CI: 0.24, 0.97; p = 0.041). There were no evident associations of taking the first dose of AL at the outlet with completed treatment in either sector or timely completion in ADDO patients, but the adjusted odds of timely completion were higher among health facility patients who took the first dose of AL at the outlet (aOR: 2.11, 95% CI: 1.46, 3.04; p < 0.001). Recalling correct instructions given by the dispenser on the AL regimen was strongly associated with both measures of adherence in public health facility and ADDO patients. In public health facilities, reporting that the dispenser used the packaging as a visual aid to explain how to take AL was also associated with timely completion (aOR = 1.77, 95% CI: 1.12, 2.80; p = 0.022). In addition, the adjusted odds of timely completion among ADDO patients who recalled being told to take the second dose after eight hours were 1.77 times that of their counterparts (95% CI: 1.12, 2.80; p = 0.015), but recalling this advice was not associated with either measure of adherence in public health facility patients. Reporting being told to complete all doses of AL even if feeling better was also not associated with either adherence measure, except for a lower adjusted odds of timely completion in public health facility patients (aOR = 0.44, 95% CI: 0.28, 0.70; p = 0.001).

## Discussion

This study indicates that patients seeking care for malaria at public health facilities and ADDOs in southern Tanzania have different characteristics, with those attending ADDOs more likely to be older, more educated, wealthier, and seeking treatment later in the day. Although similar proportions of patients from both sectors completed treatment, the proportion of patients taking each dose at the correct time (timely completion) was lower in ADDO patients. When controlling for patient characteristics, there was some evidence that the adjusted odds of completed treatment and timely completion were lower in ADDO patients compared to public health facility patients.

Completed treatment and timely completion among patients from public health facilities were 75 and 46%, respectively, comparable to other studies under real-life conditions (i.e., not clinical trials) in the public sector, which had found completed treatment verified by pill count of 64-77% and timely completion verified by pill count of 39-75% [10], including one study from Tanzania [32]. Another study from the public sector in Tanzania recently reported lower timely completion (14.9%) [35], while two other studies using different definitions and study designs found higher adherence (88.3 and 90%) [33,34]. In ADDOs, completed treatment verified by pill count was 70%, comparable to the 66% adherent by the same definition in the study by Cohen *et al.* in the private retail sector in Uganda [18].

Characteristics related to care received at the outlet differed between sectors, with health facility patients more likely to be tested for malaria at the outlet, be told their diagnosis, take the first dose of AL at the outlet, and receive advice on completing treatment even if feeling better. However, there was no difference between sectors in other advice patients reported receiving. It is possible that advice provision in Mtwara region may have been superior to that in other regions, as ADDOs in Mtwara and Lindi regions received a one-day training, including ACT treatment, in 2011. In the sector-specific models, some differences between sectors were observed in the association of these characteristics with completed treatment and timely completion, although caution in interpretation is needed given the number of comparisons made in the analyses. For example, in public health facilities, obtaining a malaria test at the outlet was not associated with completed treatment or timely completion. However, in ADDOs, patients who reported obtaining a malaria test appeared to be less adherent by both measures. One explanation for this contrast might be the status of mRDT roll out, which had occurred in public health facilities in Mtwara several months prior to the study, whereas ADDOs were not officially permitted to use mRDTs, and only 50 patients (11%) reported being tested (compared to 275 (54%) in public health facilities). The difference is not explained by reported test results, as 96% of patients reporting a malaria test in both sectors reported a positive result, though only 70% of tested health facility patients and 35% of tested ADD0 patients had a positive study mRDT at interview.

At public health facilities, taking the first dose of AL at the outlet was associated with timely completion, but not completed treatment. In ADDOs, where less than 10% of patients took the first dose at the outlet, there was no evidence of an association with either measure of adherence. Taking the first dose at the outlet might improve adherence by providing a model for patients or caregivers on how to take treatment, generating more communication with patients, or improving their confidence to complete the remaining doses at home. In addition, patients in both sectors who recalled correct instructions on how to take AL had much higher odds of completing treatment and timely completion than patients who did not recall correct instructions. This highlights the importance of clear instructions for achieving adherence [36]. A review of the previous literature shows considerable variation in factors associated with adherence to anti-malarials, but provision of better information on how to take drugs has been an important factor in more than one study [10]. Alternatively, patients who are more conscientious about their treatment may also be more likely to recall instructions. Another factor that might affect adherence but was not assessed reliably in this study is the attendance of the patient at the outlet, which is required to obtain treatment at public health facilities but not at ADDOs. Patients who received advice second-hand could be less likely to adhere. However, this should not affect the comparison between sectors, as whether or not the patient attended the outlet might lie on the causal pathway between sector and adherence, similar to the other variables related to care received at the outlet. Other factors that were not assessed, related to patient understanding of treatment or respect for dispensers, might also be important in explaining adherence.

Timely completion was 30–35 percentage points lower than completed treatment, even though timeliness of doses was based on times of day rather than exact times. While there is consensus that ACT will only be effective if taken correctly, the importance for treatment effectiveness of the recommended time intervals between doses is less clear. In this study, only 46% of public health facility patients and 35% of ADDO patients completed all doses at the recommended intervals, and there was no association between study mRDT positivity at interview (indicating malaria infection at care seeking) and adherence. While 50% of health facility patients and 28% of ADDO patients were positive by study mRDT at interview, only 22 patients overall (approximately 2%) were positive by reference blood smear, suggesting that most patients who had been malaria positive at the time of care seeking may have been treated effectively, even though only half of these had completed all doses at the correct time. However, blood smears may not capture all submicroscopic parasitaemia present at day 4, although these could lead to subsequent treatment failure [37].

The dosing regimen for AL in national guidelines states that doses should be taken at 0, 8, 24, 36, 48, and 60 hours, but for practical reasons a simpler regimen is recommended, illustrated by pictograms on packaging, which assume that patients obtain AL in the morning, take the second dose later the same day, followed by the remaining doses morning and evening for two more days [22]. More patients from ADDOs than public health facilities had obtained AL later in the day, as ADDOs had longer operating hours than public health facilities. Patients who obtained AL in the evening in the analysis for the effect of sector, and public health facility patients who obtained AL in the afternoon or evening, had lower adjusted odds of timely completion than patients obtaining AL in the morning. If eight hours after the first dose falls in the middle of the night, patients may not wake up to take the second dose, and they may be unsure when to take the remaining doses. Modified recommendations and pictograms for patients obtaining AL in the evening may be helpful in improving adherence, but this depends on establishing a clearer basis for the importance of the timing of dose intervals.

This study has several limitations. Patients may have altered their behaviour if they became aware of a potential visit or study objectives. In an attempt to prevent this, information given to dispensers was limited, visits of study research assistants to the outlets were minimized, and the study was conducted in a large number of outlets, each for a short period of time. The data presented here are also based primarily on patient self-report, which is susceptible to recall bias and social desirability bias, if patients did not remember when each dose was taken or provided the expected responses in order to avoid being seen as negligent. In addition, patients’ consultations with dispensers were intentionally not observed to avoid influencing behaviour. Instead patients’ reports of care and advice received were analysed, though recall may not have been accurate or advice of good quality. While dispensers’ characteristics and knowledge of advice to provide to patients were reported as part of the intervention study in ADDOs [31], interviews of dispensers were not conducted in health facilities, and it was therefore not possible to assess the impact of these factors across the sectors. Similarly, there may have been other important patient or care-related factors that were not assessed.

This study was conducted in the context of AFMm-subsidized ACT, and the median cost of AL in ADDOs was low (approximately $0.04 per tablet, or $0.84 for an adult equivalent treatment dose). In a setting without AL subsidies, adherence could vary. One could argue that lower adherence in ADDO patients could be a reason not to continue a subsidy of ACT in these outlets. However, the differences in adherence levels were not very large and the reasons for the differences remain unclear. Moreover, even if subsidized ACTs were not available in ADDOs (as was previously the case) patients would likely continue to seek care at these outlets, but obtain less effective anti-malarials. Thus, improving care for malaria at both ADDOs and public health facilities should be a priority.

## Conclusion

Similar proportions of patients dispensed ACT from public health facilities and ADDOs completed treatment, but the proportion with timely completion was lower in ADDO patients. Characteristics of patients obtaining ACT differed between sectors. When controlling for patient characteristics, there was some evidence that the adjusted odds of completed treatment and timely completion for ADDO patients was lower than that for public health facility patients. Further studies are necessary to understand and improve the impact of patient care on adherence, including the role of effective provision of advice.

## Additional files

Additional file 1:
**Association of patient characteristics with adherence by sector.**
Additional file 2:
**Association of factors related to care received at outlet and patient status at interview with adherence by sector.**
