# Supplementary material for: Are Tanzanian patients attending public facilities or private retailers more likely to adhere to artemisinin-based combination therapy?
Source: Malar J. 2015 Feb 19;14:87. doi: 10.1186/s12936-015-0602-x (PMC4340668; doi:10.1186/s12936-015-0602-x)
Supplement: Additional file 1: — Association of patient characteristics with adherence by sector. [file 12936_2015_602_MOESM1_ESM.docx]

**Additional file 1: Association of patient characteristics with adherence by sector**

|  | **Verified completed treatment^1^** | | | | | | **Verified timely completion^2^** | | | | | |
| --- | --- | --- | --- | --- | --- | --- | --- | --- | --- | --- | --- | --- |
|  | Public health facilities (N=572) | | | Private ADDOs (N=450) | | | Public health facilities (N=572) | | | Private ADDOs (N=450) | | |
|  | Percent adherent | Unadjusted odds ratio (95% CI) | p-value | Percent adherent | Unadjusted odds ratio (95% CI) | p value | Percent adherent | Unadjusted odds ratio (95% CI) | p-value | Percent adherent | Unadjusted odds ratio (95% CI) | p-value |
| *Sex* |  |  |  |  |  |  |  |  |  |  |  |  |
| Female (ref) | 73.5 | --- | --- | 66.0 | --- | --- | 46.7 | --- | --- | 33.3 | --- | --- |
| Male | 75.9 | 1.13  (0.74, 1.74) | 0.5 | 73.1 | 1.40  (0.88, 2.21) | 0.2 | 45.0 | 0.93  (0.70, 1.25) | 0.7 | 36.0 | 1.13  (0.81, 1.57) | 0.5 |
| *Age*^3^ |  |  |  |  |  |  |  |  |  |  |  |  |
| Under 3 years (ref) | 72.8 | --- | --- | 70.4 | --- | --- | 46.5 | --- | --- | 38.8 | --- | --- |
| 3 years to under 8 years | 76.3 | 1.20  (0.78, 1.84) | 0.4 | 66.7 | 0.84  (0.46, 1.54) | 0.6 | 45.9 | 0.98  (0.63, 1.51) | 0.9 | 29.4 | 0.66  (0.40, 1.08) | 0.095 |
| 8 years to under 12 years | 75.7 | 1.16  (0.54, 2.51) | 0.7 | 73.2 | 1.15  (0.52, 2.56) | 0.7 | 48.7 | 1.09  (0.63, 1.90) | 0.8 | 39.0 | 1.01  (0.50, 2.04) | 0.9 |
| 12 years and above | 75.4 | 1.14  (0.63, 2.06) | 0.7 | 70.4 | 1.00  (0.58, 1.72) | 0.9 | 44.3 | 0.92  (0.59, 1.43) | 0.7 | 35.0 | 0.85  (0.53, 1.37) | 0.5 |
| *Patient (or caregiver if patient below age 12) completed primary school*^4^ |  |  |  |  |  |  |  |  |  |  |  |  |
| No (ref) | 70.8 | --- | --- | 59.1 | --- | --- | 45.9 | --- | --- | 30.7 | --- | --- |
| Yes | 77.8 | 1.45  (1.03, 2.04) | 0.033 | 74.1 | 1.98  (1.27, 3.11) | 0.003 | 46.4 | 1.02  (0.75, 1.39) | 0.9 | 36.4 | 1.30  (0.84, 1.99) | 0.2 |
| *Socioeconomic status^5^* |  |  |  |  |  |  |  |  |  |  |  |  |
| 1^st^ quintile (most poor, ref) | 69.2 | --- | --- | 63.0 | --- | --- | 42.0 | --- | --- | 29.6 | --- | --- |
| 2^nd^ quintile | 70.5 | 1.06  (0.69, 1.64) | 0.8 | 57.1 | 0.78  (0.40, 1.55) | 0.5 | 44.5 | 1.11  (0.68, 1.81) | 0.7 | 25.8 | 0.83  (0.45, 1.53) | 0.6 |
| 3^rd^ quintile | 71.7 | 1.13  (0.67, 1.90) | 0.7 | 67.0 | 1.19  (0.72, 1.99) | 0.5 | 38.2 | 0.85  (0.51, 1.41) | 0.5 | 39.6 | 1.56  (0.69, 3.53) | 0.3 |
| 4^th^ quintile | 85.9 | 2.70  (1.54, 4.74) | 0.001 | 73.8 | 1.65  (0.85, 3.20) | 0.1 | 53.1 | 1.56  (0.91, 2.68) | 0.1 | 35.6 | 1.32  (0.57, 3.07) | 0.5 |
| 5^th^ quintile (least poor) | 86.4 | 2.84  (1.21, 6.67) | 0.017 | 76.4 | 1.90  (0.92, 3.93) | 0.085 | 64.3 | 2.48  (1.36, 4.52) | 0.003 | 36.6 | 1.38  (0.62, 3.05) | 0.4 |
| *Slept under a bed net the night before the interview* |  |  |  |  |  |  |  |  |  |  |  |  |
| No (ref) | 68.2 | --- | --- | 70.3 | --- | --- | 42.6 | --- | --- | 41.7 | --- | --- |
| Yes | 76.9 | 1.55  (1.16, 2.07) | 0.003 | 69.5 | 0.96  (0.63, 1.47) | 0.9 | 47.2 | 1.21  (0.81, 1.80) | 0.4 | 31.7 | 0.65  (0.45, 0.94) | 0.021 |
| *Sought care prior to attending the study outlet* |  |  |  |  |  |  |  |  |  |  |  |  |
| No (ref) | 74.4 | --- | --- | 68.8 | --- | --- | 46.6 | --- | --- | 32.6 | --- | --- |
| Yes | 75.2 | 1.05  0.73, 1.51) | 0.8 | 71.4 | 1.13  (0.71, 1.81) | 0.6 | 45.1 | 0.94  (0.66, 1.34) | 0.7 | 38.4 | 1.29  (0.87, 1.91) | 0.2 |
| *Sought care within two days of fever onset*^6^ |  |  |  |  |  |  |  |  |  |  |  |  |
| No (ref) | 71.0 | --- | --- | 66.9 | --- | --- | 36.2 | --- | --- | 32.3 | --- | --- |
| Yes | 75.6 | 1.27  (0.84, 1.91) | 0.3 | 71.0 | 1.21  (0.75, 1.92) | 0.4 | 48.8 | 1.68  (1.15, 2.46) | 0.008 | 35.8 | 1.17  (0.76, 1.79) | 0.5 |
| *Fever symptoms* |  |  |  |  |  |  |  |  |  |  |  |  |
| No (ref) | 58.8 | --- | --- | 65.0 | --- | --- | 39.4 | --- | --- | 35.0 | --- | --- |
| Yes | 75.6 | 2.16  (1.09, 4.31) | 0.028 | 70.3 | 1.27  (0.64, 2.55) | 0.5 | 46.4 | 1.33  (0.65, 2.72) | 0.4 | 34.8 | 0.99  (0.54, 1.80) | 0.9 |
| *Respiratory symptoms* |  |  |  |  |  |  |  |  |  |  |  |  |
| No (ref) | 74.9 | --- | --- | 69.7 | --- | --- | 46.6 | --- | --- | 34.2 | --- | --- |
| Yes | 72.5 | 0.88  (0.53, 1.47) | 0.6 | 70.6 | 1.04  (0.47, 2.30) | 0.9 | 42.3 | 0.84  (0.59, 1.21) | 0.4 | 41.2 | 1.35  (0.65, 2.78) | 0.4 |
| *Stomach ache* |  |  |  |  |  |  |  |  |  |  |  |  |
| No (ref) | 71.3 | --- | --- | 69.0 | --- | --- | 44.4 | --- | --- | 33.3 | --- | --- |
| Yes | 77.4 | 1.38  (0.96, 1.97) | 0.082 | 70.6 | 1.08  (0.78, 1.49) | 0.6 | 47.3 | 1.13  (0.86, 1.48) | 0.4 | 36.2 | 1.14  (0.79, 1.63) | 0.5 |
| *Fatigue* |  |  |  |  |  |  |  |  |  |  |  |  |
| No (ref) | 75.2 | --- | --- | 69.4 | --- | --- | 46.3 | --- | --- | 34.0 | --- | --- |
| Yes | 69.0 | 0.73  (0.45, 1.21) | 0.2 | 71.6 | 1.11  (0.64, 1.93) | 0.7 | 42.9 | 0.87  (0.46, 1.63) | 0.7 | 38.3 | 1.20  (0.71, 2.05) | 0.5 |
| *Joint / body pain* |  |  |  |  |  |  |  |  |  |  |  |  |
| No (ref) | 74.7 | --- | --- | 70.0 | --- | --- | 45.7 | --- | --- | 33.1 | --- | --- |
| Yes | 73.9 | 0.96  (0.56, 1.65) | 0.9 | 69.3 | 0.97  (0.65, 1.46) | 0.9 | 47.6 | 1.08  (0.71, 1.66) | 0.7 | 38.5 | 1.27  (0.80, 1.99) | 0.3 |
| *Convulsions* |  |  |  |  |  |  |  |  |  |  |  |  |
| No (ref) | 74.3 | --- | --- | 69.7 | --- | --- | 46.4 | --- | --- | 34.5 | --- | --- |
| Yes | 85.7 | 2.08  (0.44, 9.74) | 0.4 | 100^7^ | --- | --- | 28.6 | 0.46  (0.16, 1.34) | --- | 100^7^ | --- | --- |
| *Other symptoms^8^* |  |  |  |  |  |  |  |  |  |  |  |  |
| No (ref) | 74.4 | --- | --- | 70.4 | --- | --- | 45.1 | --- | --- | 34.4 |  |  |
| Yes | 75.7 | 1.07  (0.63, 1.83) | 0.8 | 64.4 | 0.76  (0.41, 1.40) | 0.4 | 52.2 | 1.33  (0.75, 2.35) | 0.3 | 38.6 | 1.20  (0.57, 2.55) | 0.6 |
| *Outlet ward* |  |  |  |  |  |  |  |  |  |  |  |  |
| Rural (ref) | 76.2 | --- | --- | 68.8 | --- | --- | 46.1 | --- | --- | 36.3 | --- | --- |
| Urban | 64.0 | 0.56  (0.26, 1.18) | 0.1 | 70.3 | 1.07  (0.63, 1.81) | 0.8 | 45.3 | 0.97  (0.51, 1.85) | 0.9 | 34.1 | 0.91  (0.52, 1.59) | 0.7 |
| *Distance from home to outlet by GPS coordinates^9^* |  |  |  |  |  |  |  |  |  |  |  |  |
| More than 2.5 km (ref) | 66.3 | --- | --- | 71.9 | --- | --- | 42.3 | --- | --- | 33.3 | --- | --- |
| 2.5 km or less | 78.1 | 1.81  (1.20, 2.75) | 0.005 | 65.8 | 0.75  (0.54, 1.06) | 0.1 | 48.1 | 1.26  (0.83, 1.92) | 0.3 | 33.8 | 1.02  (0.70, 1.50) | 0.9 |
| *Time of day drug was obtained* |  |  |  |  |  |  |  |  |  |  |  |  |
| Morning (ref) | 75.7 | --- | --- | 75.5 | --- | --- | 49.9 | --- | --- | 43.1 | --- | --- |
| Afternoon | 73.3 | 0.88  (0.50, 1.54) | 0.7 | 67.5 | 0.67  (0.40, 1.15) | 0.2 | 38.5 | 0.63  (0.43, 0.92) | 0.017 | 34.2 | 0.69  (0.41, 1.16) | 0.2 |
| Evening | 58.3 | 0.45  (0.22, 0.93) | 0.031 | 63.0 | 0.55  (0.35, 0.89) | 0.014 | 8.3 | 0.09  (0.02, 0.37) | 0.001 | 22.4 | 0.38  (0.18, 0.79) | 0.010 |
| *Time between obtaining AL and interview (hours)*^10^ |  |  |  |  |  |  |  |  |  |  |  |  |
| 60-67 | 53.1 | --- |  | 54.5 | --- | --- | 18.9 |  |  | 19.3 | --- | --- |
| 68-72 | 72.0 | 2.27  (1.20, 4.00) | 0.004 | 72.1 | 2.16  (1.25, 3.71) | 0.006 | 43.3 | 3.31  (1.42, 7.70) | 0.005 | 35.6 | 2.31  (1.19, 4.50) | 0.013 |
| 73-84 | 76.8 | 2.92  (1.47, 5.82) | 0.002 | 70.5 | 1.99  (1.08, 3.69) | 0.028 | 47.8 | 3.96  (1.70, 9.20) | 0.001 | 43.5 | 3.22  (1.62, 6.42) | 0.001 |
| 85 or more | 84.0 | 4.62  (2.00, 10.63) | <0.001 | 87.3 | 5.74  (3.14, 10.49) | <0.001 | 56.3 | 5.57  (2.26, 13.75) | <0.001 | 44.3 | 3.33  (1.66, 6.69) | 0001 |
| ^1^Patient completed all doses, verified by pill count when available. Data missing for 2 public health facility patients and 3 ADDO patients.  ^2^Patient completed each dose at correct time with the correct number of pills per dose, verified by pill count when available. Data missing for 13 public health facility patients and 10 ADDO patients.  ^3^Age categories based on recommended age breakdown for AL blister packs in Tanzania.  ^4^Caregiver education missing for five patients attending public health facilities.  ^5^Wealth quintiles pooled for public health facility and ADDO clients using principal component analysis of sampled patients based on standard Demographic and Health Survey variables. Data missing for public health facility patient.  ^6^Number of days since illness onset missing for 3 public health facility patients and 11 ADDO patients.  ^7^Analysis not possible as only 2 ADDO patients reported convulsions.  ^8^Includes dizziness, crying/fussiness, startling (kustukastuka), sleep-talking (kuweweseka), worms, fast heart rate, stays in sun, red/inflamed eyes, and sores/ulcers.  ^9^GPS data missing from 30 public health facility patients and 52 ADDO patients.  ^10^Rounded to nearest hour. Data missing for 15 public health facility patients and 6 ADDO patients. | | | | | | | | | | | | |
