# Supplementary material for: Are Tanzanian patients attending public facilities or private retailers more likely to adhere to artemisinin-based combination therapy?
Source: Malar J. 2015 Feb 19;14:87. doi: 10.1186/s12936-015-0602-x (PMC4340668; doi:10.1186/s12936-015-0602-x)
Supplement: Additional file 2: — Association of factors related to care received at outlet and patient status at interview with adherence by sector. [file 12936_2015_602_MOESM2_ESM.docx]

**Additional file 2: Association of factors related to care received at outlet and patient status at interview with adherence by sector**

|  | **Verified completed treatment^1^** | | | | | | **Verified timely completion^2^** | | | | | |
| --- | --- | --- | --- | --- | --- | --- | --- | --- | --- | --- | --- | --- |
|  | Percent adherent, Public health facilities (N=572) | Unadjusted odds ratio (95% CI) | p-value | Percent adherent, ADDOs (N=450) | Unadjusted odds ratio (95% CI) | p value | Percent adherent, Public health facilities (N=572) | Unadjusted odds ratio (95% CI) | p-value | Percent adherent, ADDOs (N=450) | Unadjusted odds ratio (95% CI) | p-value |
| *Tested for malaria at outlet* |  |  |  |  |  |  |  |  |  |  |  |  |
| No (ref) | 70.0 | --- | --- | 70.7 | --- | --- | 40.1 | --- | --- | 35.5 | --- | --- |
| Yes | 78.8 | 1.59  (1.09, 2.33) | 0.016 | 62.0 | 0.68  (0.31, 1.46) | 0.3 | 50.8 | 1.55  (1.12, 2.13) | 0.008 | 30.0 | 0.78  (0.41, 1.50) | 0.5 |
| *Told diagnosis at outlet* |  |  |  |  |  |  |  |  |  |  |  |  |
| No (ref) | 71.3 | --- | --- | 68.3 | --- | --- | 45.7 | --- | --- | 35.3 | --- | --- |
| Yes | 77.4 | 1.38  (0.83, 2.28) | 0.2 | 71.8 | 1.18  (0.76, 1.84) | 0.5 | 46.3 | 1.02  (0.68, 1.54) | 0.9 | 34.4 | 0.96  (0.70, 1.30) | 0.8 |
| *Took first dose of AL at outlet* |  |  |  |  |  |  |  |  |  |  |  |  |
| No (ref) | 73.8 | --- | --- | 68.8 | --- | --- | 40.2 | --- | --- | 34.0 | --- | --- |
| Yes | 75.8 | 1.11  (0.73, 1.70) | 0.6 | 79.1 | 1.71  (0.87, 3.39) | 0.1 | 54.4 | 1.78  (1.29, 2.45) | <0.001 | 41.9 | 1.40  (0.67, 2.90) | 0.4 |
| *Obtained correct blister pack for age^3^* |  |  |  |  |  |  |  |  |  |  |  |  |
| No (ref) | 78.3 | --- | --- | 65.8 | --- | --- | 47.8 | --- | --- | 34.2 | --- | --- |
| Yes | 73.6 | 0.77  (0.45, 1.30) | 0.3 | 70.6 | 1.25  (0.86, 1.83) | 0.3 | 45.5 | 0.91  (0.61, 1.36) | 0.7 | 34.9 | 1.03  (0.59, 1.79) | 0.9 |
| *Paid for AL* |  |  |  |  |  |  |  |  |  |  |  |  |
| No (ref) | 75.1 | --- | --- | 69.6 | --- | --- | 47.8 | --- | --- | 30.0 | --- | --- |
| Yes | 73.6 | 0.92  (0.59, 1.44) | 0.7 | 80.0 | 0.57  (0.14, 2.35) | 0.4 | 41.8 | 0.79  (0.55, 1.12) | 0.2 | 34.9 | 1.25  (0.29, 5.42) | 0.8 |
| *Recalled correct instructions given by dispenser on the number of pills per dose, number of doses, and number of days to take AL* |  |  |  |  |  |  |  |  |  |  |  |  |
| No (ref) | 58.3 | --- | --- | 55.6 | --- | --- | 22.8 | --- | --- | 22.4 | --- | --- |
| Yes | 85.0 | 4.06  (2.71, 6.09) | <0.001 | 79.4 | 3.08  (2.16, 4.41) | <0.001 | 60.9 | 5.26  (3.59, 7.71) | <0.001 | 43.3 | 2.65  (1.68, 4.19) | <0.001 |
| *Recalled that dispenser used packaging as a visual aid to explain how to take AL* |  |  |  |  |  |  |  |  |  |  |  |  |
| No (ref) | 65.4 | --- | --- | 61.6 | --- | --- | 32.1 | --- | --- | 26.0 | --- | --- |
| Yes | 76.3 | 1.70  (1.04, 2.78) | 0.036 | 71.4 | 1.56  (0.94, 2.59) | 0.090 | 48.3 | 1.98  (1.29, 3.05) | 0.002 | 36.5 | 1.64  (1.01, 2.65) | 0.046 |
| *Reported being told to take the second dose of AL eight hours after the first dose* |  |  |  |  |  |  |  |  |  |  |  |  |
| No (ref) | 67.4 | --- | --- | 59.8 | --- | --- | 41.7 | --- | --- | 24.8 | --- | --- |
| Yes | 79.8 | 1.91  (1.30, 2.80) | 0.001 | 75.6 | 2.09  (1.56, 2.80) | <0.001 | 49.1 | 1.35  (0.94, 1.92) | 0.1 | 40.5 | 2.06  (1.44, 2.94) | <0.001 |
| *Reported being told to take AL with food or milk* |  |  |  |  |  |  |  |  |  |  |  |  |
| No (ref) | 72.5 | --- | --- | 66.1 | --- | --- | 45.8 | --- | --- | 33.7 | --- | --- |
| Yes | 75.8 | 1.19  (0.72, 1.96) | 0.5 | 72.1 | 1.33  (0.91, 1.93) | 0.1 | 46.1 | 1.01  (0.65, 1.56) | 0.9 | 35.4 | 1.08  (0.69, 1.68) | 0.7 |
| *Reported being told to complete all doses of AL even if feeling better* |  |  |  |  |  |  |  |  |  |  |  |  |
| No (ref) | 66.7 | --- | --- | 63.2 | --- | --- | 46.0 | --- | --- | 28.1 | --- | --- |
| Yes | 76.9 | 1.67  (1.09, 2.56) | 0.019 | 73.6 | 1.62  (1.13, 2.34) | 0.009 | 46.1 | 1.00  (0.65, 1.55) | 0.9 | 38.6 | 1.61  (1.25, 2.06) | <0.001 |
| *Reported being told to take a replacement dose in case of vomiting within half hour of taking a dose* |  |  |  |  |  |  |  |  |  |  |  |  |
| No (ref) | 74.4 | --- | --- | 69.8 | --- | --- | 46.1 | --- | --- | 35.0 | --- | --- |
| Yes | 81.8 | 1.55  (0.37, 6.40) | 0.6 | 72.7 | 1.16  (0.40, 3.36) | 0.8 | 40.0 | 0.78  (0.19, 3.21) | 0.7 | 27.3 | 0.70  (0.19, 2.59) | 0.6 |
| *Reported being told about possible side effects* |  |  |  |  |  |  |  |  |  |  |  |  |
| No (ref) | 74.7 | --- | --- | 70.1 | --- | --- | 45.7 | --- | --- | 35.1 | --- | --- |
| Yes | 76.9 | 1.13  (0.32, 4.03) | 0.9 | 76.9 | 1.42  (0.37, 5.39) | 0.6 | 61.5 | 1.90  (0.72, 5.06) | 0.2 | 30.8 | 0.82  (0.25, 2.75) | 0.8 |
| *Reported current fever at time of interview* |  |  |  |  |  |  |  |  |  |  |  |  |
| No (ref) | 75.2 | --- | --- | 72.1 | --- | --- | 46.7 | --- | --- | 35.7 | --- | --- |
| Yes | 71.8 | 0.84  (0.53, 1.34) | 0.5 | 56.3 | 0.50  (0.27, 0.94) | 0.031 | 42.1 | 0.83  (0.55, 1.26) | 0.4 | 29.2 | 0.74  (0.40, 1.37) | 0.3 |
| *Could play or work at time of interview* |  |  |  |  |  |  |  |  |  |  |  |  |
| No (ref) | 64.3 | --- | --- | 39.4 | --- | --- | 43.9 | --- | --- | 23.5 | --- | --- |
| Yes | 75.5 | 1.71  (0.85, 3.44) | 0.1 | 72.4 | 4.04  (1.88, 8.66) | <0.001 | 46.1 | 1.09  (0.58, 2.05) | 0.8 | 35.8 | 1.81  (0.74, 4.42) | 0.2 |
| *Tested positive by mRDT at interview^4^* |  |  |  |  |  |  |  |  |  |  |  |  |
| No (ref) | 76.1 | --- | --- | 69.8 | --- | --- | 47.5 | --- | --- | 36.6 | --- | --- |
| Yes | 73.7 | 0.88  (0.51, 1.51) | 0.6 | 69.8 | 1.00  (0.69, 1.45) | 0.9 | 44.9 | 0.90  (0.57, 1.41) | 0.6 | 33.3 | 0.87  (0.60, 1.25) | 0.4 |
| *Tested positive by blood smear collected at interview^5^* |  |  |  |  |  |  |  |  |  |  |  |  |
| No (ref) | 74.6 | --- | --- | 69.8 | --- | --- | 45.3 | --- | --- | 35.6 | --- | --- |
| Yes | 75.0 | 1.02  (0.35, 2.98) | 0.9 | 66.7 | 0.87  (0.15, 4.88) | 0.9 | 56.3 | 1.56  (0.60, 4.04) | 0.4 | 33.3 | 0.91  (0.15, 5.60) | 0.9 |
| ^1^Patient completed all doses, verified by pill count when available. Data missing for 2 public health facility patients and 3 ADDO patients.  ^2^Patient completed each dose at correct time with the correct number of pills per dose, verified by pill count when available. Data missing for 13 public health facility patients and 10 ADDO patients.  ^3^Age categories based on recommended age breakdown for AL blister packs in Tanzania.  ^4^RDT data missing for 7 public health facility patients and 17 ADDO patients.  ^5^Blood smear data missing for 15 public health facility patients and 18 ADDO patients. | | | | | | | | | | | | |
